# Inhibition of bovine platelets aggregation in response to *Hyalomma anatolicum* salivary gland proteins/peptides

**DOI:** 10.14202/vetworld.2016.1264-1268

**Published:** 2016-11-17

**Authors:** Surbhi, Nirmal Sangwan, Arun K. Sangwan, Vijender Singh, Ankit Kumar

**Affiliations:** 1 Department of Veterinary Physiology and Biochemistry, Lala Lajpat Rai University of Veterinary & Animal Sciences, Hisar - 125 004, Haryana, India; 2 Department of Veterinary Parasitology, Lala Lajpat Rai University of Veterinary & Animal Sciences, Hisar - 125 004, Haryana, India; 3 Department of Veterinary Medicine, Lala Lajpat Rai University of Veterinary & Animal Sciences, Hisar - 125 004, Haryana, India

**Keywords:** anti-platelet aggregating proteins/peptides, gel filtration chromatography, *Hyalomma anatolicum* salivary gland, thrombin

## Abstract

**Aim:**

Ticks are obligate ectoparasites that have an impact on wide range of vertebrates and also act as a potential vector for the transmission of tropical theileriosis, babesiosis, etc., causing significant loss to livestock production worldwide. While feeding, they introduce their saliva containing different bioactive molecules into the host. These molecules have the capability to counteract the host hemostatic mechanism to suck host blood successfully. Therefore, the study was aimed to isolate anti-platelet aggregating peptides from salivary gland extract (SGE) of *Hyalomma anatolicum* ticks, a commonly available tick in India.

**Materials and Methods:**

Female *H. anatolicum* salivary glands were dissected out and SGE was prepared by homogenizing it in a suitable buffer under ice. Extract so obtained was fractionated by gel filtration chromatography using Sephacryl S-200 column. Total protein concentration in fractions was estimated and bovine platelets were isolated, stimulated with thrombin (positive control), treated with Gly-Pro-Arg-Pro amide (negative control) and with salivary gland fractions for identification of proteins/peptides having anti-platelet aggregating activities.

**Results:**

Proteins/peptides present in various salivary gland fractions inhibited the bovine platelet aggregation and the percent inhibition ranged between 33% and 35.8%.

**Conclusion:**

The results suggests that the fractions of *H. anatolicum* salivary glands possess thrombin-induced anti-platelet aggregating activity and which could be further exploited for raising anti-tick vaccine and also for therapeutic purpose.

## Introduction

Ticks are ectoparasites exclusively feed on host blood only. Their bite(s) results into blood loss, damage to hides and decreased milk production as the animals are under stress. They act as a leading vector for spirochetes, protozoa, viruses, and Rickettsiae as compared to any other arthropod [1], thus transmit a wide variety of pathogenic microorganisms affecting livestock, companion animals and humans. Important tick-borne diseases include babesiosis, tropical theileriosis, tick-borne encephalitis, and Lyme disease. The global economic losses by tick infestation through direct production losses and the associated cost of treatment has been estimated at US$14,000-18,000 million loss annually; while in India tick-borne diseases in livestock alone accounts for US$498.7 million loss a annum [2].

Ticks introduce their mouthparts into the host and generate a feeding cavity from which they feed on blood [3] and deposit saliva at the site of their attachment to a host to inhibit hemostasis, inflammation and innate and adaptive immune responses. Ticks need to overcome these defensive mechanisms to remain attached to the host for the entire feeding period to have successful blood meal [4]. These include direct collagen inhibitors [5], inhibitors of platelet adhesion to collagen [6], apyrases and catechol oxidases which are able to counteract the physiologic pro-hemostatic mechanisms that are triggered to avoid blood loss. Analyses of anti-platelet sialogenins may be useful tools in cell biology and may also have potential for therapeutic applications. A number of thrombin inhibitors have been isolated from ticks such as savignin from the soft ticks *Ornithodoros* *savignyi* [7] and ixin from *Ixodes ricinus* [8]. YY-39 was identified from many tick salivary glands found to inhibit adenosine diphosphate, thrombin, and TXA2 platelet aggregation [9].

Boophilin a multifunctional Kunitz protease inhibitor purified from the Midgut of *Rhipicephalus microplus* found to inhibit cathepsin G- and plasmin-induced platelet aggregation [10]. The ixodid ticks *Hyalomma anatolicum* is endemic in India infesting dairy animals responsible for transmitting *Theileria annulata* [11] causing tropical theileriosis. However, little information is available regarding anti-hemostatic substances found in salivary gland of *H. anatolicum*, the commonly available tick in India. The study was planned to isolate, fractionate the salivary gland proteins/peptides and to see the effects of proteins/peptides on bovine platelet aggregation.

## Materials and Methods

### Ethical approval

Research was conducted after due approval from Institutional Animal Ethics Committee.

### Sample collection

*H. anatolicum* female ticks were collected and identified as per the key given by Miranpuri and Gill [12].

### Tick dissection and collection of salivary glands

Ticks were washed with normal saline, immobilized individually on a Petri dish kept on ice by glue and incised along the dorsal-lateral margin using fine scalpel blade under a stereoscopic dissection microscope (Magnus MSZ-TR). Non-infected salivary glands were removed by fine tip forceps, transferred into 4-(2-hydroxyethyl)-1-piperazineethanesulfonic acid saline buffer, pH - 7.0 and stored in liquid nitrogen till analyzed.

### Salivary gland extract preparation

Hundred pairs of ticks’ salivary glands were pooled and homogenized using tissue homogenizer (T10 basic ULTRA-TURRAX^®^, India) under cold conditions. The homogenate was centrifuged at 12,000 ×*g* for 7 min at 4°C, supernatant removed, filtered through Millex-GV Syringe Filter Unit, 25 mm polyvinylidene fluoride (PVDF) 22 µm Sterile with Vent. The resulting filtrate was diluted to 2 ml with 50 mM Tris-Cl, pH 8.3 and then used for fractionation, isolation and identification of anti-platelet aggregating factors from *H. anatolicum* salivary glands.

### Isolation of anti-platelet aggregating factors from the salivary glands

The filtrate was applied to a Sephacryl S-200 gel filtration column (1 cm × 60 cm) equilibrated with 50 mM Tris-HCL, pH - 7.5 with 100 mM KCL and eluted with 40 mM Tris-Cl, pH - 7.5, fractions were collected each of 1.5 ml. The column was calibrated with molecular weight markers from Sigma (alcohol dehydrogenase - 150 kDa; albumin - 66 kDa; carbonic anhydrase - 29 kDa; cytochrome C - 12.4 kDa; and the void volume determined with Blue Dextran - 2000 kD). The approximate molecular weights of proteins were determined using a standard curve of V_e_/V_o_ against log molecular weight. Protein concentration of each fraction was estimated [13], using bovine serum albumin as standard.

### Platelets preparation

Bovine blood was collected from buffalo calves maintained at Department of Veterinary Physiology and Biochemistry, Lala Lajpat Rai University of Veterinary and Animal Sciences (LUVAS), Hisar using 0.1 M trisodium citrate as anticoagulant in 9:1 and platelet rich plasma (PRP) was obtained by centrifugation at 1000 rpm. Then, PRP was centrifuged at 4000 rpm to get the platelet pellet. The platelet pellet was washed twice with Tyrode buffer “A” (with ethylether)-N,N’-tetraacetic acid [EGTA]), and then the final pellet was resuspended in the Tyrode buffer “B” (without EGTA), in a volume adjusted to give an OD of 0.15 at 650 nm.

### Platelet aggregation assay

Effect of different isolated protein fractions on platelet aggregation was measured using ELISA plate reader at 650 nm by the method followed by Francischetti *et al*. [14] with little modification. Platelets were incubated with Gly-Pro-Arg-Pro (1 mM) amide as antagonist and isolated protein fractions for 10 min at 37°C in 96 well flat bottom plate. Then, the aggregation was initiated by adding thrombin (0.5 nM) as agonist. Changes in platelet aggregation were monitored at 650 nm at every 5 min interval for 20 min.

## Results

**Figure-1:**
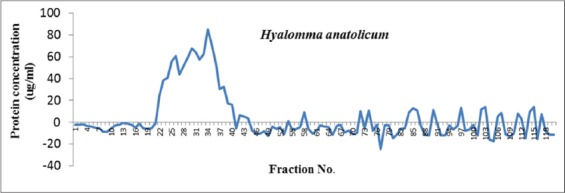
Total protein concentration of *Hyalomma anatolicum* salivary gland fractions collected by gel exclusion chromatography.

**Figure-2:**
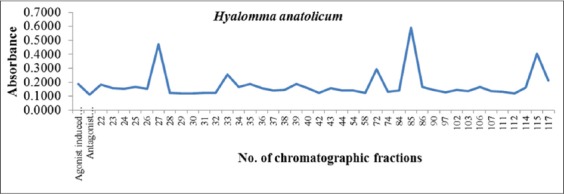
Effects of *Hyalomma anatolicum* salivary gland protein fractions on bovine platelet aggregation stimulated with thrombin.

**Figure-3:**
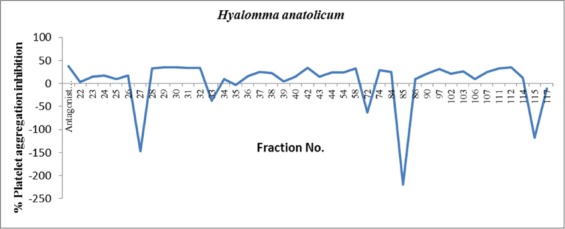
Percent platelet aggregation inhibition by *Hyalomma anatolicum* salivary gland protein fractions

**Figure-4:**
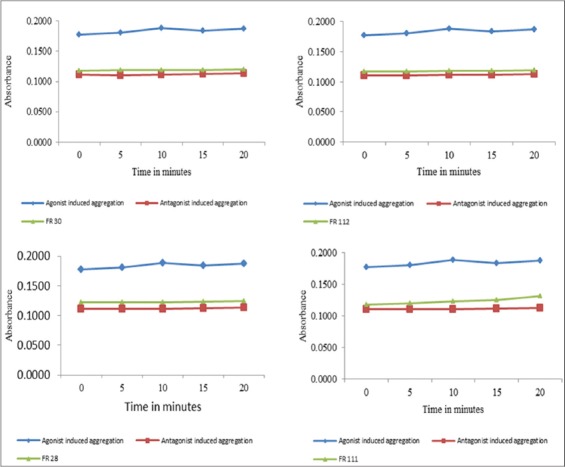
Activity of *Hyalomma anatolicum* salivary gland individual protein fractions on bovine platelet aggregation inhibition after stimulation with thrombin (0.5 nM)

A total of 120 gel filtration chromatographic fractions were collected and analyzed for total protein concentration which ranged from 0.7 to 85.2 µg/ml (Figure-1). Fractions having proteins were further analyzed for platelet aggregation inhibitory activities already stimulated with thrombin. Effects of various *H. anatolicum* salivary protein fractions on bovine platelet aggregation inhibition are shown in Figure-2. It was observed that fraction nos. 28, 29, 30, 31, 32, 42, 58, 111, and 112 having platelet aggregation inhibition activities nearly similar to that of antagonist-induced aggregation inhibition and was much higher as compared to agonist-induced platelet aggregation. The total protein concentration in these above-mentioned fractions was found to be 51.5, 58.8, 67.7, 63.9, 57.5, 6.2, 9.1, 7.9 and 3.3 µg/ml, respectively. Percent platelet aggregation inhibition by *H. anatolicum* salivary gland fractions ranged from 33% to 35.8%. The inhibition was comparatively less due to proteins in various fractions as compared to that of antagonist peptide where 38.3% inhibition was observed (Figure-3). The effects of individual protein fractions having the anti-platelet aggregating activities are shown in Figure-4. It was reported that various proteins present in different fractions behaved differently in terms of platelet aggregation inhibition when compared to that of agonist thrombin-stimulated platelet aggregation and antagonist-induced platelet inhibition. Fraction no. 30 and 112 had nearly similar activities to that of antagonist while the fraction no. 28 and 111 activity had slightly less inhibitory activity. The potency for the antithrombin effect of the tested compounds at 35.8% inhibition was the strongest for fraction no. 112 (3.3 µg protein/ml).

## Discussion

*H. anatolicum* salivary gland selected fractions were found to inhibit bovine platelet aggregation which was stimulated by thrombin. When compared with the peptide (Gly-Pro-Arg-Pro) known to have platelet aggregation inhibitory activities, some of the fractions showed comparable inhibitory effect than the known inhibitory peptide while others showed the slightly less effects to that of inhibitory peptide. This inhibitory peptide is known to inhibit fibrin polymerization while thrombin is known to induce platelet aggregation via interaction among thrombin exosites and the substrates on platelet membrane, namely, proteins platelet activating receptors-1 (PAR-1) and PAR-4 [15,16]. Similarly, Nienaber *et al*. [7] reported the thrombin-induced aggregation inhibitor savignin from the soft tick *O. savignyi* while Hoffmann *et al*. [8] isolated ixin platelet inhibitor from *I. ricinus*. Ibelli *et al*. [17] characterizes *Ixodes scapularis* (Ixsc) IxscS-1E1, a blood meal-induced serine protease inhibitor from Ixsc tick saliva and was found to inhibit 23.4% thrombin-induced aggregation. De Queiroz *et al*. [18] isolated BmooAi, from *Bothrops moojeni* that inhibited platelet aggregation. Sanchez *et al*. [19] reported BarI, isolated from the venom of *Bothrops barnetti* found to dissolves fibrin clots made either from purified fibrinogen or from whole blood. In *H. anatolicm* maximum inhibitory activity was found to be in range of 33-35.8% in different fractions. In this study, the inhibitory effects of proteins/peptides in various fractions can be either due to inhibition of fibrin polymerization or due to inhibition of action of thrombin exosites, PAR-1 and PAR-4 receptors which are mainly found in activated platelets for platelet plug formation at the site of vascular injury and thereby preventing the conversion of fibrinogen to fibrin.

## Conclusion

A significant amount of platelet aggregation inhibitory proteins/peptides was found in salivary gland of *Hyalomma anatolicum* tick whose mechanism of action needs to be further exploited for raising anti-tick vaccines as well as for therapeutic purposes.

## Authors’ Contributions

NS designed the experiment, data analyses, preparation and final corrections of the manuscript. S and AKS collected the sample. AKS identified the tick species and contributed in the dissection of ticks. S performed the dissection of ticks, wet lab analyses, and data analyses. VS and AK helped during analysis. All authors participated in manuscript preparation. All authors read and approved the final manuscript.
